# Dietary Supplementation with Fumaric Acid Improves Growth Performance in Nile Tilapia Juveniles

**DOI:** 10.3390/ani12010008

**Published:** 2021-12-21

**Authors:** Suzane C. V. das Neves, Suzianny M. B. C. da Silva, Gisely K. A. Costa, Eudes S. Correia, Alexandre L. Santos, Lilian C. R. da Silva, Álvaro J. A. Bicudo

**Affiliations:** 1 Departamento de Pesca e Aquicultura, Universidade Federal Rural de Pernambuco, Dois Irmãos, Recife 52171-900, PE, Brazil; suvarela.pesca@hotmail.com (S.C.V.d.N.); suzianny.silva@ufrpe.br (S.M.B.C.d.S.); gisely.costa01@gmail.com (G.K.A.C.); escorreia@uol.com.br (E.S.C.); 2 Departamento de Zootecnia, Universidade Federal do Paraná—Rua Pioneiro, 2153, Palotina 85950-000, PR, Brazil; aleseur@gmail.com (A.L.S.); lcrsilva@ufpr.br (L.C.R.d.S.)

**Keywords:** fish nutrition, feed additives, growth promoters, sustainable aquaculture, acidifiers

## Abstract

**Simple Summary:**

The benefits of dietary supplementation with organic acids are commonly known in farm animals, especially swine and poultry, but these compounds are rarely exploited as additives to aquafeeds. Fumaric acid (FA) and its salts are among the most used organic acids in terrestrial animal feeds, but research with fish diets is limited. This study evaluated the inclusion of FA (0, 5, 10, 15, 20, and 30 g/kg) in the diets of Nile tilapia juveniles, and showed that supplementation improved growth, feed efficiency, protein use, and intestinal villi morphometry. Additionally, the population of undesirable intestinal bacteria was reduced (Gram-negative) or eliminated after 28 days (Enterobacteriaceae) in fish fed fumaric acid diets. Therefore, this study supports the addition of fumaric acid to the diet as a reliable strategy to improve the production and intestinal microbiota health of Nile tilapia juveniles.

**Abstract:**

Organic acids have recently been identified as promising replacements for antibiotics in aquafeeds that promote fish growth and feed efficiency. This study evaluated the inclusion of fumaric acid (FA; 0, 5, 10, 15, 20, and 30 g/kg) in diets (350 g/kg CP; 3.4 kcal digestible energy/g) of Nile tilapia juveniles. Fish (average weight 1.7 ± 0.1 g) were distributed in three 40 L aquaria per treatment (13 fish/aquaria) in a completely randomized design. Over 35 days, the fish received the experimental diets three times daily to apparent satiety. The maximum weight gain, feed efficiency ratio, and protein efficiency ratio were recorded in fish supplemented with 14–15 g/kg FA. After 28 days, Enterobacteriaceae was registered only in the gut of tilapia without FA augmentation. Gram-negative bacteria in the fish gut decreased (*p* < 0.05) in fish receiving 17 g/kg of dietary FA, increased after this level. The intestinal villi height and width were affected (*p* < 0.05) by FA levels and feeding time. Thus, inclusion of 15 g/kg of FA was effective in promoting growth, improving intestinal morphometry, and decreasing negative gut bacteria of Nile tilapia juveniles after 35 days.

## 1. Introduction

The growth of the aquaculture industry and the consequent intensification of production systems has increased the health challenges for cultivated fish, with direct consequences on their intestinal microbiome, which has an important role in host health and nutritional metabolism [1]. There is increasing pressure for more sustainable aquaculture systems, where the reduction or, preferably, elimination of antibiotics from the production cycle is desired to avoid developing cross-resistance of pathogens to antibiotics and their residues. Thus, the search for alternative feed additives (e.g., pre- and probiotics, essential oils, enzymes, and organic acids) to modulate and improve the gut health of fish has been the focus of researchers in recent years.

The benefits of dietary supplementation with short-chain organic acids and their salts, also called acidifiers, on growth, feed efficiency, and health of terrestrial livestock, have been evident for decades [2,3]. Acidifiers affect the intestinal tract in two ways: (i) by reducing pH in the stomach and small intestine, and (ii) by acid dissociation in the bacterial cell and salt anion accumulation that inhibits microbial growth [4]. Despite this, the intensification of studies on organic acids with aquatic animals began only at the end of the 2000–2009 decade [5], although the first study evaluating different acids in fish diets was performed by Rungruangsak and Utne in 1981 [6].

Several studies have reported the benefits of supplementation with various organic acids or their salts in fish diets [7,8,9,10], although negative results have also been documented [11,12,13]. In general, these contradictory reports relate to differences between species and/or rearing conditions [5]. Although Ng and Koh [5] stated that propionic acid, followed by fumaric, formic, and lactic acid, and their salts are the most used in animal feeds, research on fumaric acid in aquafeeds has been limited. To our knowledge, fumaric acid was evaluated only as a single additive in African catfish *Claria gariepinus* diets [14,15] or as part of dietary organic acid blends for yellow catfish *Pelteobagrus fulvidraco* [16]. In contrast, fumaric acid is one of the most widely studied organic acids for terrestrial animal feeds, especially pigs [3] and broiler chickens [2].

Fumaric is a weak organic acid with four carbons (C_4_H_4_O_4_) that is generally stable at room temperature, has a medium solubility in water, and is odorless, nontoxic, and naturally found in plants and fungi [17]. In animal metabolism, fumaric salts are generated during the decomposition of aspartate, phenylalanine, and tyrosine in the ornithine cycle and during purine synthesis. However, when orally ingested or generated from the citric acid cycle, fumaric acid is involved in cellular ATP production [4]. Additionally, fumaric acid has antimicrobial action related to its lipophilic feature and high dissociation capacity (pKa). When crossing the bacterial cellular membrane and reducing the cytoplasmic pH, it alters bacterial metabolism and enzyme activities, inhibiting growth or inducing cell death [18].

Therefore, the aim of this study was to evaluate the dietary supplementation of fumaric acid in aquafeeds based on productive performance, intestinal microbiota effects, and villi morphometry of Nile tilapia (*Oreochromis niloticus*) juveniles.

## 2. Materials and Methods

### 2.1. Experimental Diets

A basal diet (350 g/kg CP) was formulated and supplemented with fumaric acid at 0, 5, 10, 15, 20, and 30 g/kg of diet (Table 1). Fumaric acid (Merck, Germany) was added at the expense of cellulose in a 1:1 ratio. All feedstuffs were ground (0.8 mm), weighed, mixed, moistened with 250 g/kg of distilled water, and pelletized using a meat mincer. Three-millimeter pellets were dried in an air circulation oven at 55 °C for 24 h. After processing, the pellets were ground and screened to granule sizes proportional to the mouth of the fish. Thereafter, all diets were refrigerated (4–6 °C) throughout the experimental period. The fumaric acid content of all experimental diets was analyzed (Table 2) according to the standard protocol [19].

### 2.2. Fish and General Procedures

Male Nile tilapia juveniles were provided by the Reference Center for Aquaculture and Fishery Resources (Porto Real do Colégio, AL, Brazil) of the Companhia de Desenvolvimento do Vale do São Francisco e Parnaíba (CODEVASF [(São Francisco and Parnaíba Valley Development Company)). Juveniles were fed a commercial diet (350 g/kg of crude protein) three times daily (8:00, 12:00, and 16:00 h) until apparent satiety for seven days prior to the experiment for acclimation to the experimental conditions. After this period, fish were weighed (1.7 ± 0.1 g) and randomly stocked in 40 L aquaria (13 fish/per tank) in a completely randomized design (*n* = 3). All aquaria had supplementary aeration, temperature control, and biological filters. The aquaria were siphoned daily to remove feces and any uneaten feed, and up to 15% of the total volume of water was replaced. Tilapia juveniles were fed the experimental diets for 35 days using the same method as described for the acclimation period.

Dissolved oxygen (6.7 ± 0.2 mg/L) and water temperature (27.5 ± 0.3 °C were monitored daily using a YSI Model 55 m (YSI Industries, Yellow Springs, OH, USA), and pH (7.5 ± 0.2) was monitored with an electronic pH meter (AK90, AKSO, São Leopoldo, RS, Brazil). Total ammonium (0.33 ± 0.123 mg/L) and nitrite (0.25 ± 0 mg/L) were evaluated weekly. All water parameters remained within the range considered adequate for Nile tilapia [21].

### 2.3. Microbiological and Histological Sampling and Processing

Weekly (days 7, 14, 21, 28, and 35 of the trial), four fish per treatment were fasted for 24 h, anesthetized by immersion in benzocaine solution (100 mg/L), and sacrificed by cutting the spinal cord. Each fish was then surface-sterilized with a 70% ethanol solution to ensure aseptic conditions for necropsy. The abdomen was opened with disinfected tools, and the entire intestinal tract was exteriorized. A 3 cm sample of the proximal intestine was gently rinsed by flushing with a 0.85% sterile saline solution to remove the digestive content. The sample was then mixed with 1 mL of 0.85% sterile saline solution, followed by successive dilutions (10^−1^ to 10^−5^) in saline solution. A 100 μL aliquot was inoculated in duplicate using the spread plate technique. The selective media used were MacConkey agar (Gram-negative) and eosin methylene blue agar (Enterobacteriaceae). Plates were incubated at 30 °C for 24 h, and colonies were counted using a colony counter. An additional sample of the proximal intestine (±1 cm in length) of each fish was collected for histological analysis. The samples were fixed by immersion in Bouin’s solution for 12 h, dehydrated in graded ethanol concentrations, immersed in xylol, and embedded in paraffin wax. After processing, the tissues were sectioned (5 μm) and stained with hematoxylin and eosin. The intestine samples were photographed with a Canon PowerShot SX530 HS digital camera coupled to an Olympus Bx 41 microscope. The images were processed and analyzed using ImageJ software. Measurements (height and width of intestinal villi) were performed for all villi observed from three random and non-consecutive fields of each sample.

### 2.4. Proximate and Chemical Composition Analyses

The chemical composition of the experimental diets was determined according to the standard recommendations of the Association of Official Analytical Chemists [19]. Moisture was analyzed by oven-drying samples (105 °C) to constant weight; crude protein was determined using the Kjeldahl method; crude lipid using the Soxhlet method; ash by burning in a muffle furnace at 550 °C for 24 h; calcium using an atomic absorption spectrophotometer; phosphorus spectrophotometrically using the molybdovanadate method, and fumaric acid by high-performance liquid chromatography (HPLC). All analyses were performed by a certified laboratory (CBO Análises Laboratoriais, Valinhos, São Paulo, Brazil).

### 2.5. Calculations and Statistical Procedures

The following performance parameters were calculated: weight gain (WG = final weight − initial weight ÷ initial weight × 100), feed efficiency ratio (FER = weight gain (g) ÷ dry feed intake (g)), and protein efficiency ratio (PER = weight gain (g) ÷ total protein intake).

The data were previously tested for normality (Shapiro–Wilk test) and homoscedasticity of variances (Bartlett’s test). Due to the continuous nature of the treatments, a polynomial single and multiple regression analysis was then performed to determine the optimal dietary fumaric acid supplementation level.

## 3. Results

**Figure 1:**
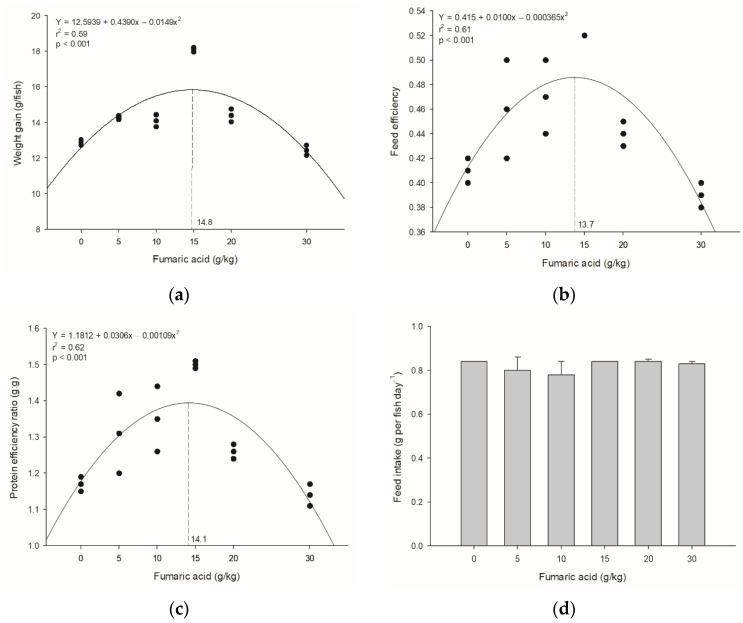
Relationship between productive performance parameters of Nile tilapia juveniles and dietary fumaric acid levels after 35 days: (**a**) weight gain; (**b**) feed efficiency ratio; (**c**) protein efficiency ratio and (**d**) feed intake. The dashed line indicates the level of fumaric acid supplementation in Nile tilapia diets for maximum response.

The survival rate during the trial was 100%, and all experimental diets were well accepted by the fish. The dietary fumaric acid level for maximum weight gain (Figure 1a), feed efficiency (Figure 1b), and the protein efficiency ratio (Figure 1c) of Nile tilapia juveniles was estimated to be approximately 14–15 g/kg. Feed intake in fish was similar (*p* > 0.05) regardless of fumaric acid levels in the diets (Figure 1d).

**Figure 2:**
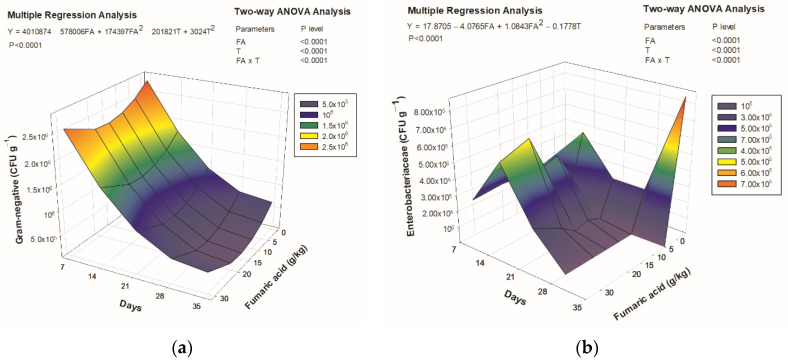
Relationship between bacteria counts in the gut of Nile tilapia juveniles and dietary fumaric acid levels through 35 days: (**a**) Gram negative bacteria; (**b**) Enterobacteriaceae. FA: fumaric acid level (g/kg); T: time (days).

The interaction between fumaric acid levels and experimental time was significant (*p* < 0.05) for the total intestinal microbiota and villi measurements. The lowest total Gram-negative bacteria was recorded in fish fed with 17 g/kg of dietary fumaric acid, as estimated by regression analysis, and showed an increasing trend after this level in the final trial. Gram-negative bacteria decreased in fish fed all diets (Figure 2a). After 28 days of the trial, Enterobacteriaceae was not detected in Nile tilapia fed diets with fumaric acid, while it remained in fish fed the control diet throughout the experimental period (Figure 2b).

**Figure 3:**
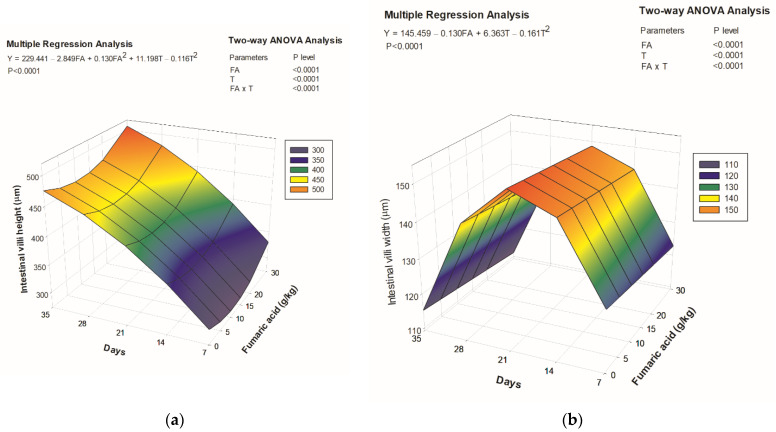
Relationship between intestinal villi morphometry of Nile tilapia juveniles and dietary fumaric acid levels through 35 days: (**a**) intestinal villi height; (**b**) intestinal villi width. FA: fumaric acid level (g/kg); T: time (days).

**Figure 4:**
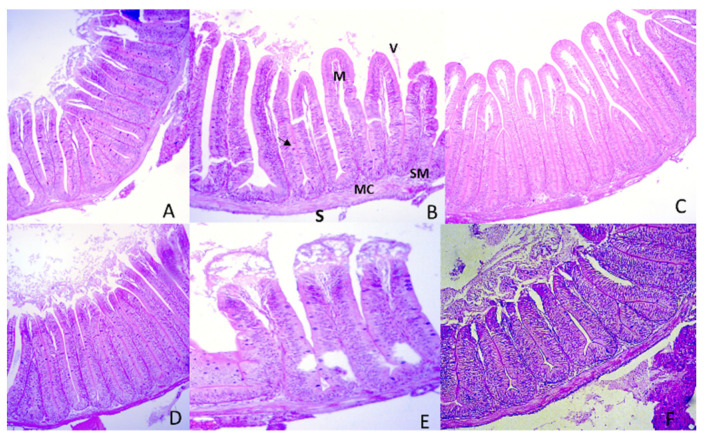
Photomicrograph of proximal intestine of Nile tilapia juveniles fed diets supplemented with fumaric acid levels of 0 g/kg (**A**), 5 g/kg (**B**), 10 g/kg (**C**), 15 g/kg (**D**), 20 g/kg (**E**) and 30 g/kg (**F**). In B, villi (V), mucosal epithelium (M), submucosa layer (SM), muscular layer (MC), serous layer (S) and goblet cell (arrow). HE staining. Bar = 200 µm.

Fumaric acid supplementation up to 11 g/kg initially decreased intestinal villi height; however, from this point onwards, the villi increased with fumaric acid supplementation up to 30 g/kg until the end of the trial (Figure 3a). In contrast, dietary fumaric acid supplementation decreased the villi width linearly (*p* < 0.05) throughout the trial; the maximum values were observed up to 21 days of the experiment, decreasing after this time (Figure 3b). The pictures of intestinal villi of Nile tilapia from each treatment are shown in Figure 4.

## 4. Discussion

Growth performance is the key factor in determining the economic benefits of fish production. In the present study, dietary supplementation with fumaric acid clearly improved the growth performance, feed efficiency, and protein efficiency ratio of Nile tilapia juveniles. The greatest difference was found for weight gain, with values increasing proportionally to fumaric acid supplementation of up to 14–15 g/kg, followed by a reduction thereafter. Previously, only Omosowone et al. [14] reported an improvement in growth, feed efficiency, and protein efficiency ratio of African catfish juveniles fed up to 1 g/kg fumaric acid diets. After eight weeks, 0.5 g/kg of malic acid diets increased weight gain and feed efficiency of Nile tilapia, but no additional gains in fish were recorded with up to 8 g/kg of supplementation with that acid [22]. The benefits of dietary supplementation of several organic acids in a pure form, such as salts or blends in aquafeeds, have been reported for many fish species [5]. These gains are usually a result of a complex group of factors such as the pH reduction in the gastrointestinal tract favoring the activity of digestive enzymes [6], and consequently the digestibility of nutrients [10]; antimicrobial activity of organic acids; and protective effects on intestinal epithelium, regardless of the intrinsic properties of the organic acid evaluated [23]. This indicates that it is necessary to determine the optimum dietary supplementation levels of fumaric acid for different fish species to maximize the benefits of this feed additive.

Dietary supplementation greater than 15 g/kg of fumaric acid tended to decrease productive performance as well as feed and protein use of Nile tilapia juveniles. This decrease in growth, feed, and protein use was also observed in African catfish fed diets with greater than 10 g/kg of fumaric acid [14]. The negative impact of organic acid supplementation on fish growth can be attributed to a reduction in feed intake due to acidification of diets, which renders them less palatable [24]. However, the feed intake of fumaric acid diets by Nile tilapia was similar to that of the non-supplemented diet, despite fumaric acid having a tart flavor [17]. In fact, tilapia has been shown to be able to adjust the ingestion of diets with a high range of supplementation of several organic acids or their salts, such as malic [9] and potassium diformate [12].

Fumaric acid originates mainly from the oxidation of succinate and is converted to malic acid in the tricarboxylic acid cycle. In pigs, fumaric acid is an energy source readily available for use by the intestinal mucosa, promoting a faster recovery of gastrointestinal cells and increased absorptive surface of the gut [3]. This is a plausible hypothesis to explain the increase in the height and width of intestinal villi in Nile tilapia fed fumaric acid-supplemented diets. Nile tilapia juveniles given malic acid supplemented diets showed higher whole body, muscle, and liver lipids, a common response to increased availability of dietary energy [25]. Additionally, the antimicrobial activity of organic acids favors the development of non-pathogenic microorganisms, forming a protective barrier that assists in the development of the villi and enables greater absorption and utilization of nutrients; thus, the improvement of growth, feed, and protein use in Nile tilapia is a result of a multifactorial set of benefits provided by fumaric acid supplementation.

Positive results of fumaric acid-supplemented diets up to 15 g/kg are clearly shown in the present study. However, higher inclusion levels of this organic acid led to decreased performance parameters in Nile tilapia juveniles. The effects of overdoses of organic acids (>10 g/kg) in aquafeeds have rarely been studied, and deserve further research. For example, clinical signs of liver damage (necrosis, sinusoid disorganization, and hemorrhage) were more apparent in Nile tilapia fingerlings fed sodium citrate-supplemented diets (20 and 40 g/kg), as were decreased growth and feed efficiency [13]. In the present study, the height and width of intestinal villi slightly decreased with fumaric acid supplementation, and increased when more than 11 g/kg fumaric acid was added. Huan et al. [10] also registered an increase in villi height of Nile tilapia diets with 0.9 g/kg of a microencapsulated organic acid salt blend. The positive effects of organic acid blends on villi morphometry can be potentiated when associated with other substances, such as essential oils [8]. However, intestinal villi height and width were similar between Nile tilapia fed diets supplemented with or without organic acids [10]. Omosowone et al. [15] reported a slight loss of intestinal microvilli and desquamation of the mucosal epithelium of African catfish fed 10 and 15 g/kg of fumaric acid. Moreover, the same authors identified anaplasia and necrosis in the intestinal villi of *C. gariepinus* fed diets with a 20 g/kg concentration. Additionally, there was a decrease in the width of villi of fish after 21 days of fumaric acid supplementation. Despite this, no pathological signs were observed in the intestinal villi of Nile tilapia regardless of fumaric acid supplementation in the present study.

The antimicrobial effectiveness of organic acids depends on the dissociation constant (pKa), with higher values indicating greater effectiveness. Fumaric acid has a high dissociation potential, with values between 3.02 and 4.76 [5], enabling it to cross the bacterial cell membrane. In the specific case of Gram-negative bacteria, this effect is enhanced due to the thin cellular wall formed by a single layer or few layers of glycan-peptide and an outer membrane separated by the periplasmatic space [26].

Colonization and establishment constitute a complex process that depends on factors such as the microbial composition of the water, the diet provided, development stage, stress level, and the environment. This occurs because the intestine has a wide variety of aerobic and anaerobic microorganisms [27,28,29,30], which would explain the late colonization observed in the present study.

The literature offers few studies involving diets supplemented with organic acids, particularly fumaric acid, and the evaluation of the bacterial community in the gastrointestinal tract of tilapia. In vitro trials reported that the use of certain organic acids, such as formic, acetic, propionic, and butyric, inhibited the growth of the pathogenic *Vibrio harveyi*, with the strongest inhibitory effect achieved with formic acid [31].

Among the bacterial agents in aquatic ecosystems, Gram-negative Enterobacteriaceae can be found in the skin, gills, and intestines of fish [31]. Some species, such as *Enterobacter* spp., *Klebsiella* spp., *Escherichia coli*, *Proteus* spp., *Serratia marcescens*, *Salmonella* spp., and *Citrobacter* spp., [31] are important opportunistic pathogens that can cause intestinal disease [32,33,34]. In the present study, the addition of fumaric acid to the diet inhibited the growth of enterobacteria in the intestine beginning on Day 28, independent of the supplementation level, thereby constituting a viable strategy for the exclusion of potentially pathogenic bacteria from the animal microbiota.

Hybrid tilapia fed a diet supplemented with potassium diformate at a proportion of 0.6% exhibited an increase in weight gain as well as a change in the intestinal bacterial community [12]. Kluge et al. [35] also investigated the gastrointestinal microflora of piglets for a period of 35 days and found a reduction in total aerobic and anaerobic bacteria as well as lactic acid-producing and Gram-negative bacteria.

## 5. Conclusions

Dietary inclusion of 15 g/kg of fumaric acid improved growth performance and proved to be effective in reducing intestinal Gram-negative bacteria in Nile tilapia juveniles after 35 days.

## Figures and Tables

**Table 1:** Formulation and chemical composition (wet basis) of basal diet.

Ingredient	Content (g/kg)
Soybean meal	344.4
Fish meal	200.0
Corn	295.4
Corn starch	100.0
Soybean oil	20.0
Fumaric acid	0.00
Cellulose	30
Vitamin-mineral premix ^1^	10.0
BHT ^2^	0.20
Analyzed chemical composition	
Moisture	76.6
Crude protein	353.0
Crude lipid	115.4
Ash	81.9
Calcium	15.5
Phosphorus	11.2
Digestible energy (kcal/g) ^3^	3.4

^1^ Guaranteed levels (kg/product): vit. A—1,000,000 IU; vit. D3—312,500 IU; vit. E—18,750 IU; vit. K3—1250 mg; vit. B1 (thiamine)—2500 mg; vit. B2 (riboflavin)—2500 mg; vit. B6 (pyridoxine)—1875 mg; vit. B12—4 mg; vit. C—31,250 mg; nicotinic acid—12,500 mg; calcium pantothenate—6250 mg; biotin—125 mg; folic acid—750 mg; choline—50,000 mg; inositol—12,500 mg; iron sulfate—6250 mg; copper sulfate—625 mg; zinc sulfate—6250 mg; manganese sulfate—1875 mg; sodium selenite—13 mg; calcium iodate—63 mg and cobalt sulfate—13 mg. ^2^ BHT = butylated hydroxytoluene. ^3^ Calculated according to Furuya et al. [20].

**Table 2:** Fumaric acid content of experimental diets.

	Acid Fumaric Levels (g/kg)					
Supplemented	0	5	10	15	20	30
Analyzed	0.43	5.6	9.1	15.7	21.2	30.5

## Data Availability

The datasets generated during and analyzed during the current study are available from the corresponding author upon reasonable request.
